# Continued weakening of the equatorial Pacific upwelling annual cycle in CMIP5 future projections

**DOI:** 10.1038/s41598-022-19874-2

**Published:** 2022-09-16

**Authors:** Li-Chiao Wang, Thi Lan Dao, Jia-Yuh Yu

**Affiliations:** 1 Department of Atmospheric Sciences, National Central University, Taoyuan, Taiwan; 2 School of Geography, Earth and Atmospheric Sciences and ARC Centre of Excellence for Climate Extremes, The University of Melbourne, Melbourne, VIC Australia

**Keywords:** Climate sciences, Ocean sciences

## Abstract

This study explores the dynamics of the equatorial Pacific upwelling annual cycle under global warming using the Coupled Model Intercomparison Project Phase 5 (CMIP5) simulations. Through a linear-weighted theory developed recently, the theoretical upwelling annual cycles under global warming helped reasonably characterize the patterns of the original upwelling annual cycles simulated in CMIP5 models; however, an apparent weakening in magnitude as compared to that during the present stage was observed. To verify the above, we divided 90-year outputs in the CMIP5 future projections into three 30-year windows and set side by side. The long-term evolution of the upwelling annual cycle reconfirmed an overall weakening tendency in the entire equatorial Pacific. Moreover, the weakening of the Ekman upwelling could most likely be attributed to the meridional surface wind stress divergence, while the gradually smoothing inclination in the overall equatorial thermocline depth was responsible for the weakening of the wave upwelling. The weakening of the wave upwelling in the east and the Ekman upwelling in the west jointly contributed to the gradual weakening of the equatorial Pacific upwelling annual cycle. The above projected changes are robust among the 19 chosen CMIP5 models. Equatorial upwelling largely influences the sea surface temperature, associated atmosphere–ocean interactions, and convection and precipitation in tropical areas; hence, a continuous weakening of the upwelling annual cycle over the equatorial Pacific Ocean could likely affect the major climate phenomena variability with strong seasonal-locking characteristics by modifying the background strength at their peak phases in the future. The theoretical results can provide us the equatorial upwelling annual cycle patterns based on the Ekman and wave dynamics, which would be a strong tool for our investigations on the climate variability under global warming.

## Introduction

The equatorial upwelling plays an essential role in the evolution of the cold tongue and can be hardly ignored through involving ocean dynamics adjustment^1,2^. Having strong links to the cold tongue development, the equatorial upwelling and its annual cycle deeply impact the entire tropical climate system^3,4^. The contribution of equatorial upwelling is also important to marine ecosystems besides considering its importance to climatic aspects. The rise and retreat of the equatorial upwelling are closely linked to nutrient concentrations and the subsequent primary and secondary productivity^5^. Hence, monitoring equatorial upwelling undoubtedly benefits scientific development and human progress.

Due to the limitations of observational data considering oceanic circulation, it has been challenging to comprehensively understand equatorial upwelling. Nevertheless, considerable efforts have been exerted to analyze temporal variations and the spatial distributions of trade winds, the sea surface height, ocean currents, and sea surface temperature (SST) in tropical areas to better understand equatorial dynamics^2,6–11^. The equatorial upwelling is believed to have mainly resulted from the Ekman processes driven by local winds^8,10,11^. Giordani and Caniaux^7^ reported that equatorial upwelling in the Atlantic was closely related to the imbalance between circulations and pressure fields, and turbulent momentum flux. A recent study developed a linear-weighted theory to characterize the annual cycle of equatorial upwelling based on the Zebiak–Cane (ZC) ocean model^12^. They demonstrated that two important mechanisms jointly contributed to the equatorial upwelling annual cycle: (1) the Ekman upwelling directly driven by winds; and (2) the wave upwelling associated with vertical displacements of thermocline remotely induced by winds^13–15^.

Based on their findings, we deliberated how the equatorial Pacific upwelling annual cycle could change, and how the impacts due to the Ekman and wave processes could vary under global warming. The mean equatorial upwelling was found to weaken in the equatorial Pacific^2,16^, while it strengthened in the Atlantic^17^. However, long-term variations in the equatorial upwelling annual cycle are still questionable. The equatorial upwelling annual cycle is not only important toward the cold tongue evolution and the marine ecosystem but also toward climate variabilities with strong seasonal-locking characteristics, e.g., the Pacific/North-Atlantic (PNA) and the El Niño Southern Oscillation (ENSO) Pattern^18,19^. Fluctuations in the equatorial upwelling annual cycle greatly impact the background SST, deep convections, and the air-sea interactions during the peak phase of climate variability; moreover, this could further affect their intensities and frequencies. As such, clarifying the long-term variations in the equatorial upwelling annual cycle is necessary to better understand how interannual climate variabilities could evolve in a future warmer climate. Here, the dynamics of upwelling annual cycle under global warming in equatorial Pacific was investigated along with an analysis considering the newly-developed theory. The causes of the behaviors of the equatorial upwelling annual cycle have also been thoroughly discussed.

The remainder of this study is outlined as follows. Dataset and methodology are presented in “Results” section. “Discussion and conclusions” section discusses future projections of the equatorial Pacific upwelling annual cycle based on the Coupled Model Intercomparison Project Phase 5 (CMIP5) simulations. Long-term variations in the equatorial Pacific upwelling annual cycle under global warming were also examined in the “Discussion and conclusions” section. The major findings are summarized in “Methods” section.

## Results

The performance of CMIP5 datasets in reproducing the equatorial upwelling annual cycle in both Atlantic and Pacific basins has been examined in recent literatures. Wang et al.^13^ showed that CMIP5 simulations can represent well the key features of equatorial upwelling annual cycle in the Atlantic Ocean compared to ORA-S3 observations. Wang and Yu^15^ also pointed out that the theoretical and original upwelling annual cycles in the equatorial Pacific are generally consistent in observations as well as CMIP5 simulations. Because the data period and the number of selected CMIP5 models in this study are slightly different from those in Wang and Yu^15^, the performance of CMIP5 was re-examined in supplementary material (Fig. S2). Despite some amplitude discrepancies, the simulated original and theoretical upwellings still reproduce key features of the observed ones. In addition, the weighted theory works well in identifying the equatorial Pacific upwelling annual cycle in observations as well as CMIP5 simulations, which is consistent with Wang and Yu^15^. Thus, this study continued to utilize CMIP5 models to investigate the potential changes of equatorial Pacific upwelling annual cycle under global warming.

### Simulated upwelling annual cycles

**Figure 1:**
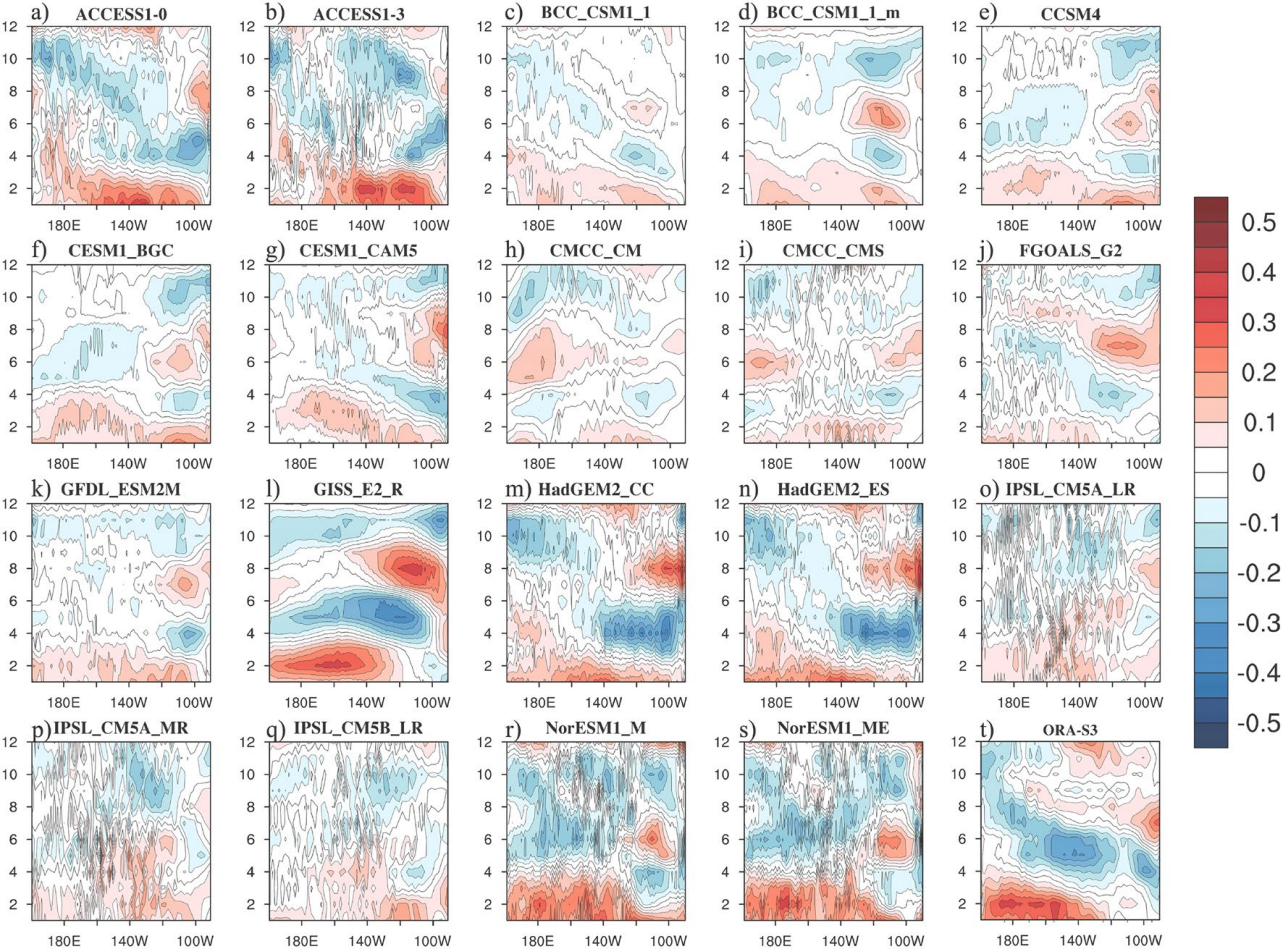
The upwelling annual cycles of the equatorial Pacific (averaged over 3°N–3°S; units: m/day) originally simulated by 19 CMIP5 models (**a**–**s**) and from observation (**t**) in the present stage during 1976–2005. The maps were generated with NCAR Command Language (NCL) Version 6.6.2 (http://www.ncl.ucar.edu/).

**Figure 2:**
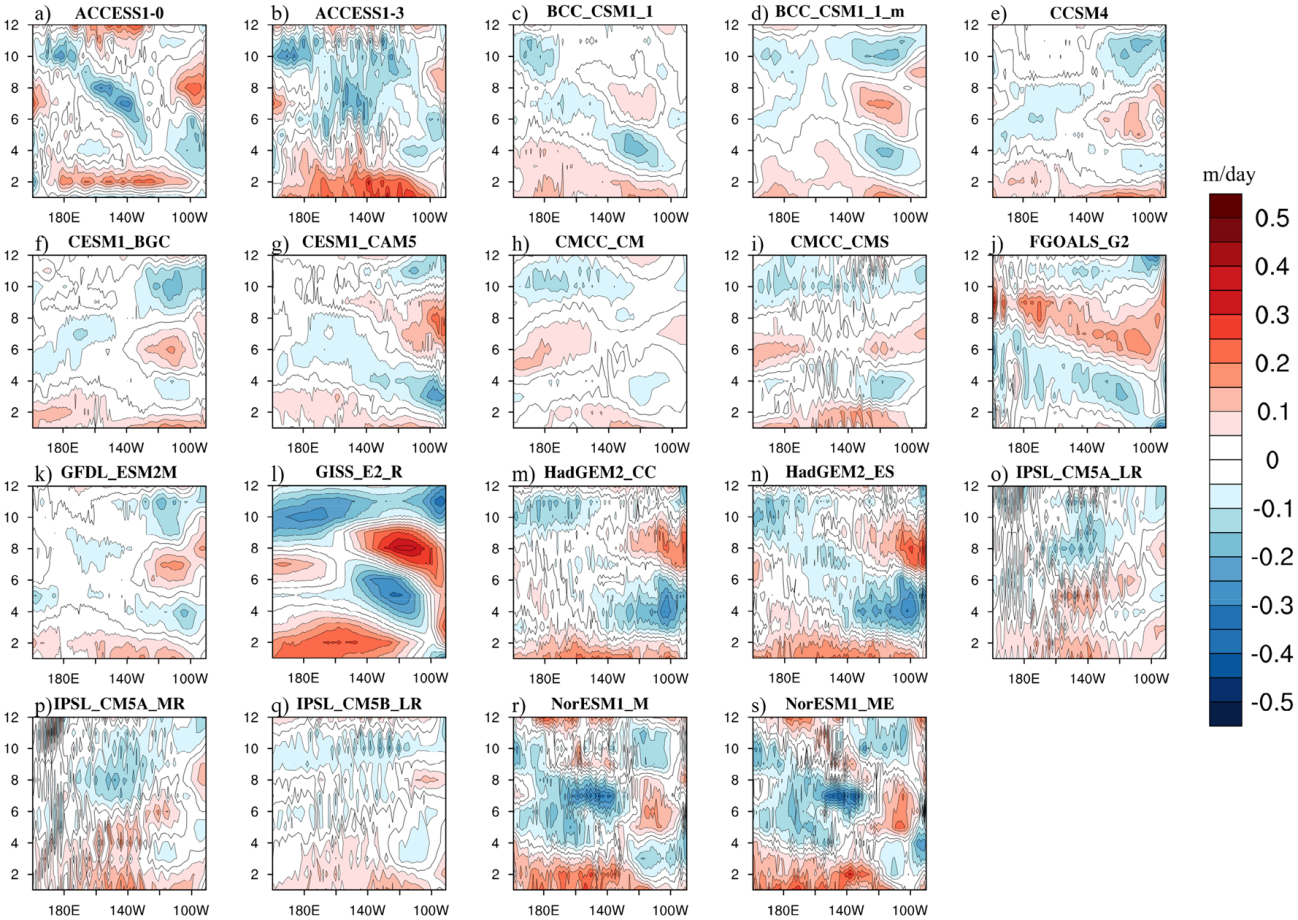
The upwelling annual cycles of the equatorial Pacific (averaged over 3°N–3°S; units: m/day) originally simulated by 19CMIP5 models in the future projections during 2071–2100. The maps were generated with NCAR Command Language (NCL) Version 6.6.2 (http://www.ncl.ucar.edu/).

Before applying the weighted theory to analyze the upwelling annual cycles under global warming, we first explored the originally-simulated upwelling annual cycle patterns from the observation and originally yielded by each model for the present stage from 1976 to 2005 (Fig. 1). The observed equatorial upwelling annual cycle is characterized by a strong component occupying all over the year in the central Pacific and a semi-annual component in the eastern Pacific. In the central basin, the downwelling (negative value) peaks around May–June, while the upwelling (positive value) occurs at the beginning and the end of year. Despite of different used period, these findings are consistent with Wang and Yu^15^, indicating the robustness of the results from ORA-S3 observational data. In the present stage of the CMIP5 simulations, the amplitudes of the upwelling annual cycles simulated by the 19 models appeared to be slightly uneven. For instance, some models (including (a) ACCESS1-0, (b) ACCESS1-3, (l) GISS-E2-R, (m) HadGem2-CC and (n) HadGEM2-ES) simulated relatively strong upwellings, and some models simulated relatively weak values in the eastern Pacific (including (c) BCC-CSM1-1 and (h) CMCC-CM). Such inter-model discrepancy in amplitudes of upwelling annual cycles is most likely resulted from the biased equatorial wind stress forcing or thermocline annual variations^13–15^. Previous studies have discussed similar dynamics that the weaker equatorial wind stress forcing might result in a flanker thermocline, weaker upwelling and SST variability^20,21^. Simulations of the semi-annual signal in the equatorial central to eastern Pacific varies among 19 CMIP5 models. For example, some models (including (a) ACCESS1-0, (b) ACCESS1-3, (m) HadGEM2-CC, and (n) HadGEM2_ES) can simulate quite well the upwelling semi-annual signals, while other models (including (c) BCC-CSM1-1, (f) CESM1-BGC, (o) IPSL-CM5A-LR, (p) IPSL-CM5A-MR, (q) IPSL-CM5B-LR, (r) NorESM1-M, and (s) NorESM1-ME) show relatively weak simulations. The difference in the semi-annual signal simulations from 19 CMIP5 models could be responsible for the weaker representative of semi-annual cycle signal in the central and eastern Pacific from the ensemble mean (Fig. S2e). It is very possible that in those models, the ocean models did not respond well under the atmospheric forcing. The simulated upwelling annual cycles in most models were predominated by a semi-annual harmonic signal over the eastern Pacific, which enhanced around the boreal winter and summer seasons and weakened over the rest of the year. Another evident pattern was the weakening sign west of 140°W starting from spring to summer (Fig. 1). In the future projections, the pattern and phases yielded by each model were similar to that in the present stage; however, the overall amplitude weakened (Fig. 2). The difference of original upwelling between present stage and future projection from individual models presented in Supplementary Materials further confirmed the weakened amplitude of the equatorial Pacific upwelling under global warming (Fig. S2). The potential causes are discussed in the subsequent section.

### Theoretical upwelling annual cycles

**Figure 3:**
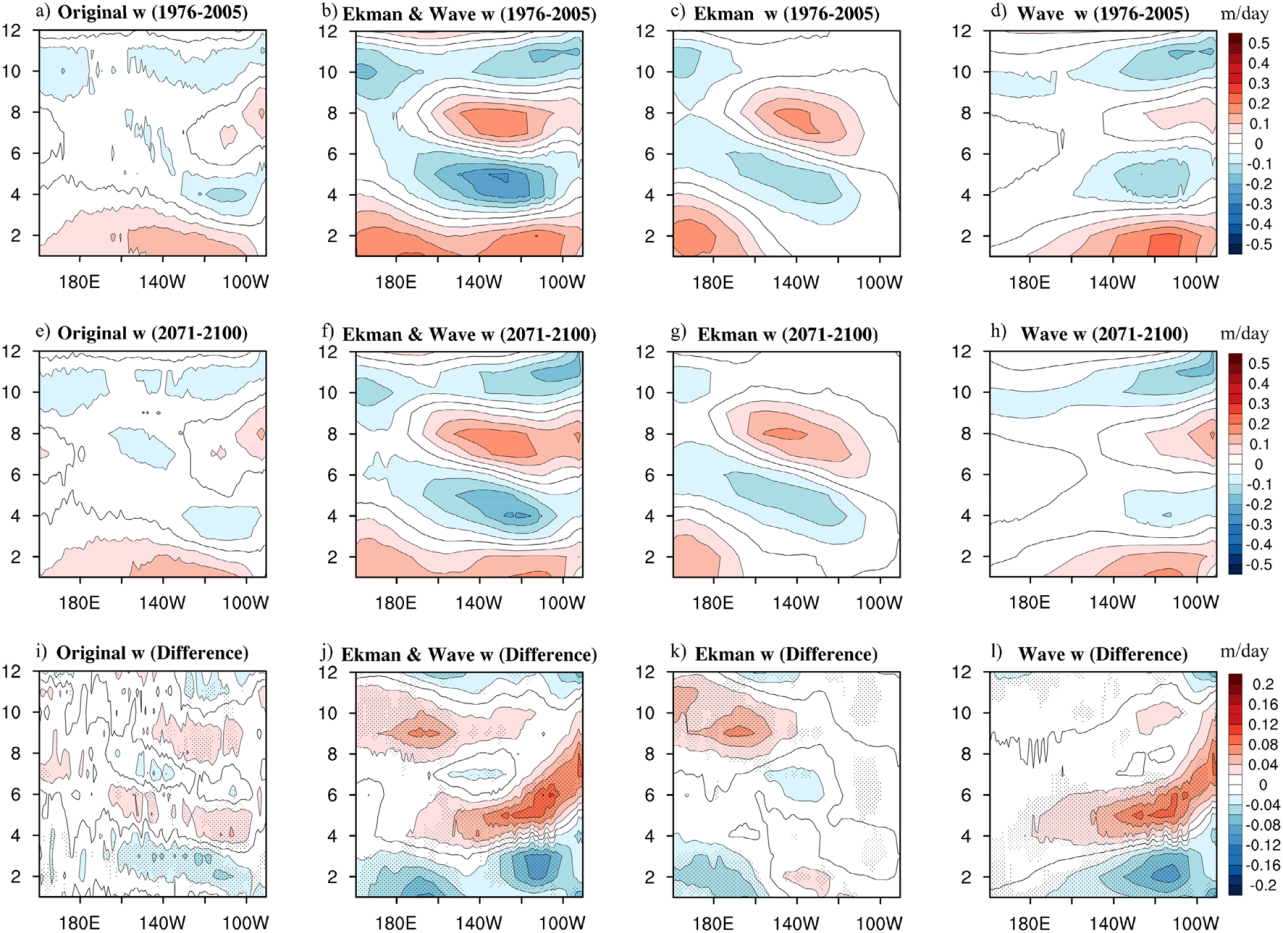
The annual cycles of the original, theoretical, Ekman, and wave upwelling in the equatorial Pacific for the (**a**–**d**) present stage, (**e**–**h**) future projections, and (**i**–**l**) the difference between the present stage and the future projections derived via the multi-model ensemble of the 19 CMIP5 simulations (averaged over 3°N–3°S; units: m/day). Black dots indicate locations over where more than 70% of models producing the same sign of equatorial upwelling difference between present and future simulations. The maps were generated with NCAR Command Language (NCL) Version 6.6.2 (http://www.ncl.ucar.edu/).

The weighted theory was applied to present simulations and the future projections to highlight the contrast in the dynamics of the upwelling annual cycles between different stages (Fig. 3). In the present stage, the original upwelling (Fig. 3a), which was the ensemble of the 19 models simulations as in Fig. 1, exhibited some common features of the equatorial Pacific upwelling annual cycle in the CMIP5 simulations: a basin-wide amplification from January to April, a semi-annual harmonic signal east of 140°W, and a large-scale weakening west of 140°W starting from April till the end of the year. These evident characteristics were generally reproduced by the theoretical upwelling (Fig. 3b); moreover, the time-longitude evolution map between the theoretical and original upwelling reaches 0.72. Although the semi-annual harmonic signal appeared to be overestimated and shifting westward^15^, the high pattern correlation indicated that the weighted theory could help reasonably characterize the key features of the original upwelling annual cycle. Hence, the weighted theory helps us discern the dynamics of the equatorial Pacific upwelling annual cycles and decompose it into the essential controls considering the Ekman upwelling (Fig. 3c) and the wave upwelling (Fig. 3d). Through these patterns, we could say that in the original upwelling, the semi-annual harmonic signal confined at the eastern Pacific was most likely attributable to the wave upwelling; while the weakening pattern west of 140°W was mainly contributed by the Ekman upwelling. Notably, the basin-wide amplifying pattern at the beginning of the year appeared to be shared between the Ekman and wave upwellings. The pattern correlation between the original and the wave (Ekman) upwellings was 0.76 (0.33). The correlation coefficients between the original upwelling and the theoretical, wave and Ekman upwellings are all significant at 95% level according to the Student’s t test.

The upwelling annual cycle in the future projection (Fig. 3e–h) demonstrated a similar pattern to that of the present stage, implying that the key features prevailed. The pattern correlation between the original and theoretically reconstructed equatorial upwelling dropped slightly to 0.68; moreover, the amplitude noticeably weakened. In the original upwelling (Fig. 3e), the original basin-wide enhancing pattern at the beginning of the year and the weakening pattern west of 140°W starting from April till the end of the year were both apparently weaker. The semi-annual harmonic signal at the eastern Pacific also slightly weakened at both the positive and negative phases. Such changes were also observed in the theoretical upwelling under global warming (Fig. 3f). As decomposed by the weighted theory, these changes could be traced back to the Ekman upwelling (Fig. 3g) and the wave upwelling (Fig. 3h). The Ekman upwelling was most likely responsible for the retreat of the enhancing pattern at the beginning of the year and the weakening pattern for the remainder of the year, which were mainly located west of 140°W (Fig. 3g). In contrast, the wave upwelling demonstrated a dominant contribution to the retreat of the semi-annual harmonic signal at the eastern Pacific (Fig. 3h). The pattern correlation between the original and the wave (Ekman) upwelling in the future projection reached 0.8 (0.25), indicating that the contribution of the wave upwelling to the entire equatorial upwelling slightly increased from the present stage to the future projection. Similar as in CIMIP5 present-stage simulation, the correlation coefficients between the original upwelling and the theoretical, wave and Ekman upwellings in future projection are also significant at 95% level.

To obtain better contrast and gain an insight into the variations in the upwelling annual cycle dynamics evolving from the present to the future, the difference between future projections and present stage (future minus present) derived via the multi-model ensemble of the 19 CMIP5 simulations is shown (Fig. 3i–l). This indicates that a weakening of the semi-annual harmonic signal at the eastern Pacific in the original upwelling (Fig. 3i) could be mainly attributed to variations in the wave upwelling (Fig. 3l). The impact of the wave upwelling was mainly observed east of 140°W. Conversely, the impact of the Ekman upwelling was mainly observed west of 140°W (Fig. 3k). The retreat of both, the enhancing pattern by April and the weakening pattern starting from June could help reasonably explain variations in the original upwelling in the western Pacific (Fig. 3i). Considering both, the Ekman and the wave upwelling (Fig. 3j) again suggest that each process alone is inadequate to characterize all the key features of the original upwelling annual cycles. Both, the Ekman and wave processes are required for accurately presenting the original upwelling annual cycles. The intermodel consistency is greater than 70% in both original and theoretical upwellings, the western (eastern) part of Ekman (wave) upwelling, suggesting that the weakening signals of the equatorial Pacific upwelling annual cycle are robust among the selected models.

**Figure 4:**
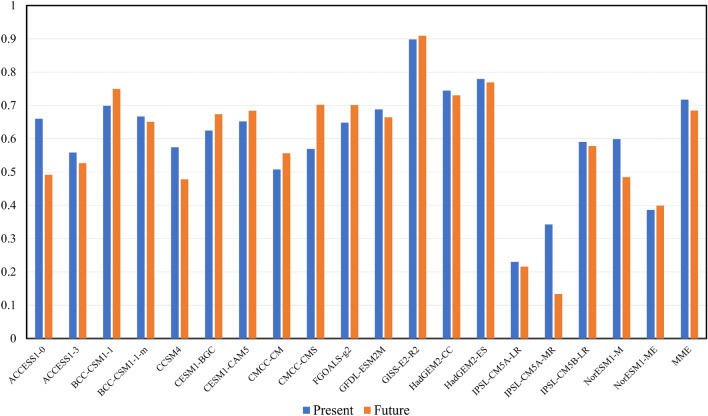
A bar chart listing correlations between the original and theoretical values of the equatorial upwelling annual cycles based on the 19 CMIP5 models. The figure was generated with NCAR Command Language (NCL) Version 6.6.2 (http://www.ncl.ucar.edu/).

To evaluate how the weighted theory works in the future projections, Fig. 4 shows the pattern correlations between the original and theoretical upwellings of the 19 CMIP5 models for the present stage (blue bars) and the future projections (orange bars). The pattern correlations yielded by most of the 19 models were > 0.6. The ensemble also attained a pattern correlation of 0.72 and 0.68 for the present stage and future projection, respectively, suggesting that most models sufficiently simulated the equatorial Pacific upwelling annual cycle. More importantly, the correlation in each model did not apparently fluctuate from the present to the future, further confirming the stability of the weighted theory. This implied that even under significant variations in the warming scenarios, the weighted theory could reasonably help characterize the main features of equatorial upwellings simulated in the CMIP5 models.

### Long-term evolution of the upwelling annual cycles in future projections

To thoroughly analyze how the upwelling annual cycles evolve under representative concentration pathway (RCP) 8.5 warming scenario, the future projections (2011–2100) were separated into three 30-year windows (2011–2040, 2041–2070, and 2071–2100), hereafter referred to as P1, P2, and P3, respectively; the three time-horizons were compared to analyze the long-term evolution of upwelling annual cycles in the CMIP5 future projections.

**Figure 5:**
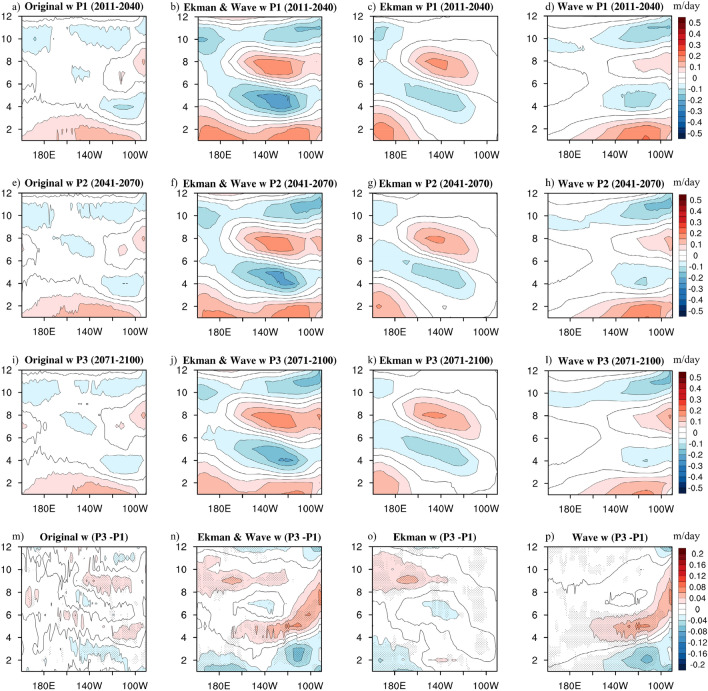
The annual cycles of the original, theoretical, Ekman, and wave upwelling in the equatorial Pacific for the (**a**–**d**) P1, (**e**–**h**) P2, (**i**–**l**) P3, and (**m**–**p**) the difference between P3 and P1 (P3–P1) via the multi-model ensemble of the 19 CMIP5 simulations (averaged over 3°N–3°S; units: m/day). Black dots indicate locations where more than 70% of models producing the same sign of equatorial upwelling difference between present and future simulations. The maps were generated with NCAR Command Language (NCL) Version 6.6.2 (http://www.ncl.ucar.edu/).

The weighted theory was applied to the projections of P1, P2, and P3, respectively. It provided further insights on the gradual change in the upwelling annual cycle dynamics considering the CMIP5 future projections (Fig. 5). For P1 (Fig. 5a–d), the theoretical upwelling (Fig. 5b) generally reflected the key features of the original upwelling (Fig. 5a): a basin-wide enhancement from January to April, a large-scale weakening in the western Pacific starting from April till the year-end, and a semi-annual harmonic signal over the central-eastern Pacific. The amplitude of the theoretical upwelling was slightly overestimated as compared to that of the original upwelling, and the eastern semi-annual harmonic signal shifted slightly westward; however, the substantial pattern that was being obtained helped us analyze the essential controls of the equatorial upwelling annual cycles. The primary characteristics of the theoretical upwelling, which reasonably helped characterize the original upwelling, appeared to be dominated by the wave (Ekman) upwelling in the east (west). The semi-annual harmonic signal confined in the central-eastern Pacific most likely resulted from the wave upwelling (Fig. 5d); moreover, the weakening pattern in the western Pacific was attributable to the Ekman upwelling (Fig. 5c). Moreover, the basin-wide enhancing pattern from January to April was seemingly attributable to both the Ekman and the wave upwelling.

The upwelling annual cycle patterns in P2 (Fig. 5e–h) and P3 (Fig. 5i–l) demonstrated that the gradual variations evolving from P1 to P2 to P3 were consistent: the basin-wide original and theoretical upwelling demonstrated an overall weakening tendency, which was also observed in the Ekman and wave upwelling. To identify the development of this gradual change over the 90-year future projections, the difference between the last and first 30 years (P3-P1) was displayed in Fig. 5m–p. First, the long-term change over the future 90 years in the original upwelling (Fig. 5a, e, i) can be dynamically-analyzed by considering the theoretical upwelling (Fig. 5b, f, j): both, the basin-wide positive pattern at the beginning of the year and the negative pattern in the western Pacific over the remainder of the year in P1 apparently weakened. Based on the phases of the semi-annual harmonic signal confined in the eastern Pacific in P1, this semi-annual signal also became irregular and weakened when evolving to P3.

Subsequently, considering the clarified pattern of the theoretical upwelling, we could reasonably link it to the Ekman upwelling (Fig. 5o) and wave upwelling (Fig. 5p), respectively. In the central-eastern Pacific, the weakened semi-annual signal is believed to have resulted from the wave upwelling; its evolution from P1 to P3 demonstrated that this semi-annual harmonic signal in the central-eastern basin significantly weakened in the first-half of the year and became slightly amplified at the end of the year according to its original phasing (Fig. 5d, h, l). Conversely, in the original/theoretical upwelling annual cycle, the weakening of the negative signal (then positive in P3-P1) in the western Pacific, from the middle till the end of the year, is attributable to the Ekman upwelling (Fig. 5c, g, k). This weakening tendency expanded eastward (~ 140°W). Another evident characteristic was the weakening of the original basin-wide positive signal at the beginning of the year (Fig. 5m, n). The difference map (P3-P1) displays an overall weakening tendency (based on their original phases) considering the entire theoretical upwelling annual cycle (Fig. 5n). This evolvement has been reasonably demonstrated to be jointly attributable to the Ekman upwelling in the west (Fig. 5o) and the wave upwelling in the east (Fig. 5p) considering the 90-year future projections. A high consistency among models regarding the long-term evolution of the upwelling annual cycle can be observed (Fig. 5m–p), suggesting a robust weakening tendency in the entire equatorial Pacific during 2011–2100 future projections.

### Dynamics of the Ekman/wave upwelling in the future projections

The Ekman upwelling was calculated from the surface wind stress following the formulation of the ZC ocean model, while the wave upwelling was obtained by considering the vertical displacement tendency of the thermocline depth. Hence, to further analyze the effect of equatorial air-sea dynamics on the Ekman and wave upwelling during the 90-year future projections, the evolution of the equatorial thermocline depth and surface wind stress was investigated.

**Figure 6:**
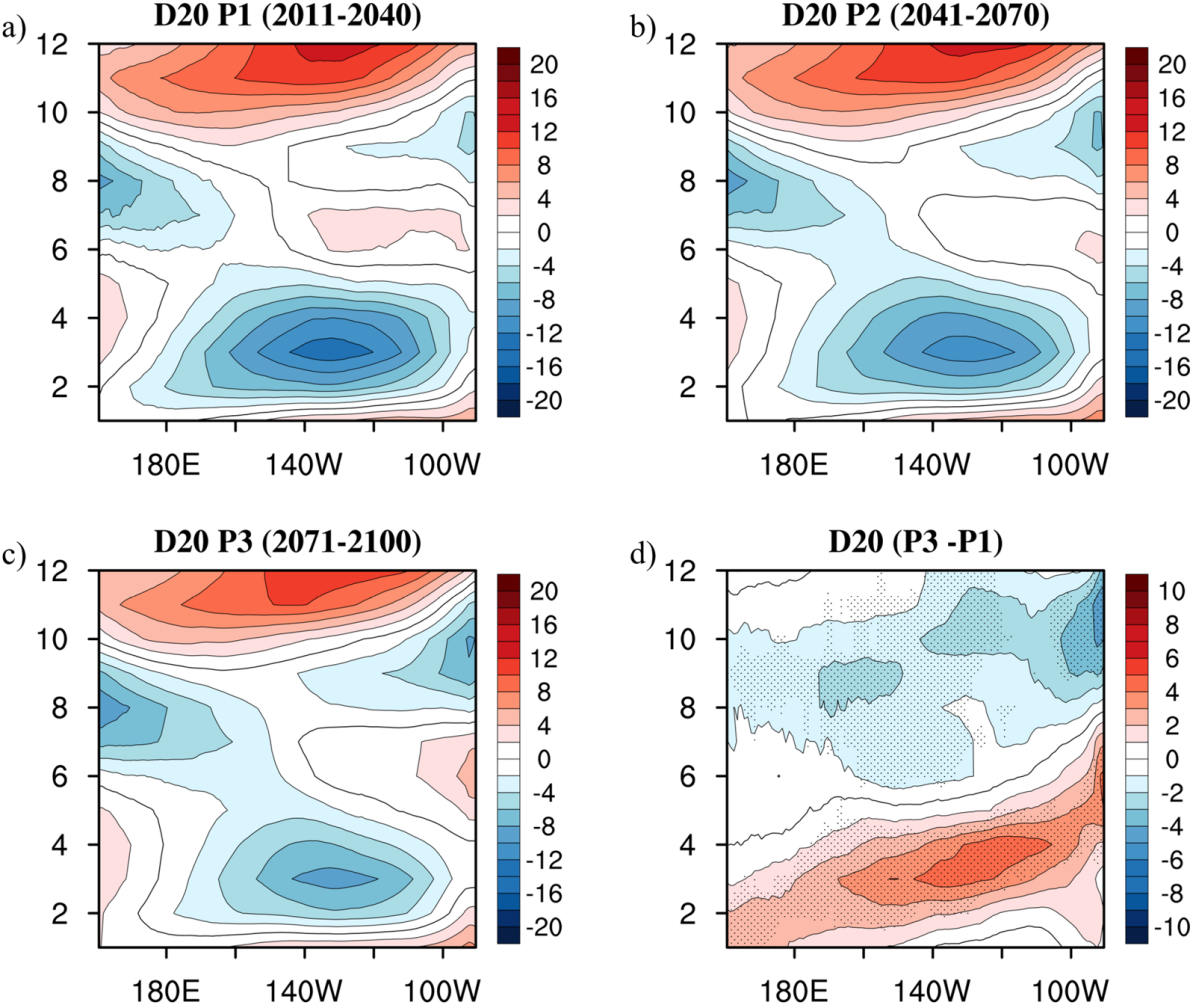
The equatorial Pacific annual cycles (averaged over 3°N–3°S) of the thermocline (meters) considering (**a**) P1, (**b**) P2, (**c**) P3, and (**d**) the difference between P3 and P1. Black dots indicate locations where more than 70% of models producing the same sign of thermocline difference between present and future simulations. The maps were generated with NCAR Command Language (NCL) Version 6.6.2 (http://www.ncl.ucar.edu/).

Figure 6a–c exhibits the annual evolution of the equatorial thermocline depth during P1, P2, and P3. Considering these 90-year future projections, it was observed that both the negative pattern expanding from January to April and the positive pattern at the end of the year west of 100°W gradually weakened. Conversely, the relatively short-scale positive/negative variations east of 100°W kept amplifying during these years. In other words, the annual fluctuations in the equatorial thermocline depth gradually smoothed in the basin-wide Pacific but slightly strengthened at the eastern boundary. The difference with a widespread agreement (greater than 70%) across the chosen models in the evolution of the thermocline depth between P3 and P1 (Fig. 6d) vividly illustrated a pattern that was in a 90-degree phase quadrature of the wave upwelling difference map (Fig. 5p). This evidently suggests that a gradual change in the wave upwelling over the CMIP5 future projections resulted from a change in the equatorial thermocline depth.

**Figure 7:**
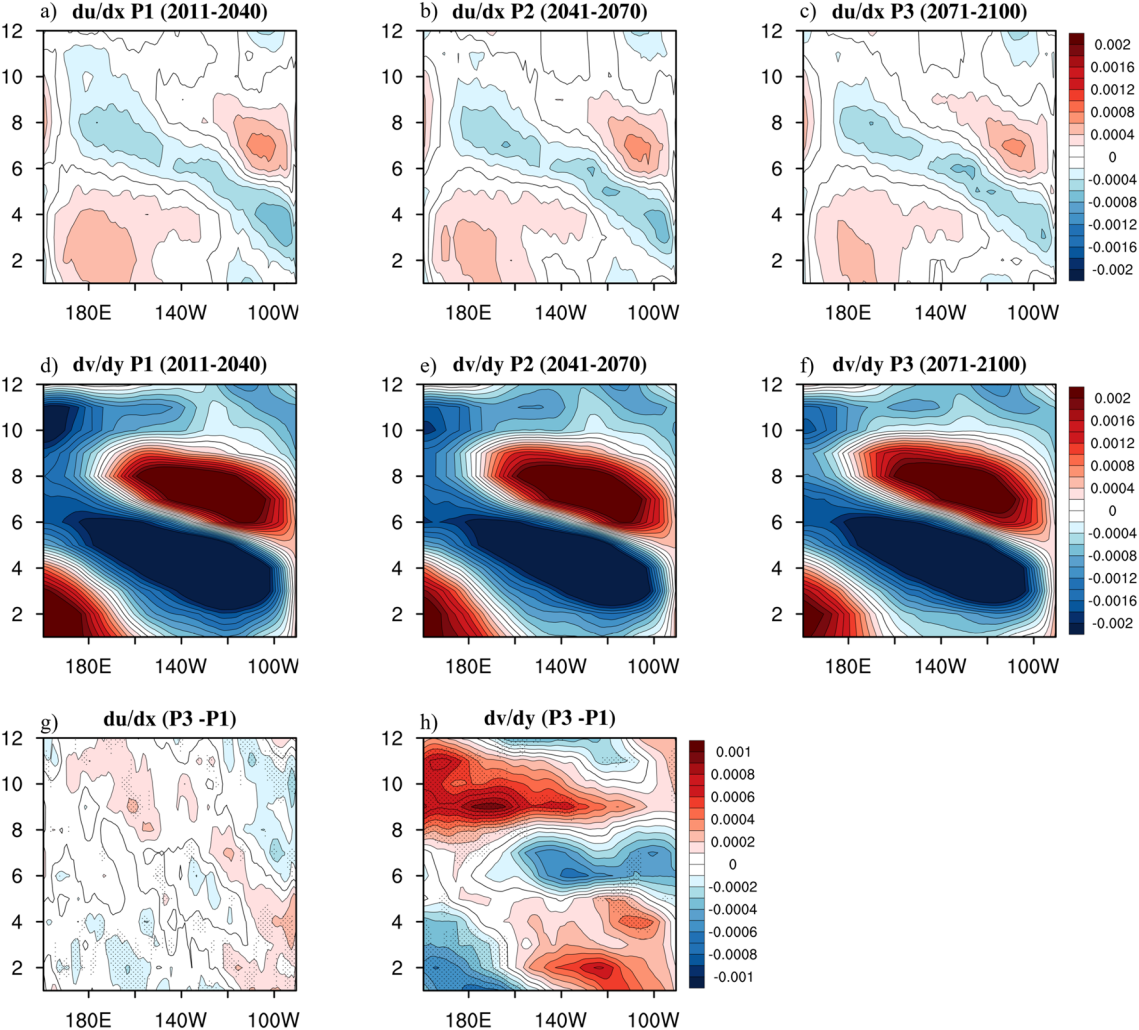
The equatorial Pacific annual cycles (averaged over 3°N–3°S) considering the (**a**–**c**) zonal and (**d**–**f**) meridional surface wind stress divergence during P1 to P3 and (**g**–**h**) the difference between P3 and P1. Black dots indicate locations where more than 70% of models producing the same sign of wind stress divergence difference between present and future simulation. The maps were generated with NCAR Command Language (NCL) Version 6.6.2 (http://www.ncl.ucar.edu/).

To explore where the gradual change in the Ekman upwelling originated from during these 90 years, the annual evolution of surface wind stress divergence in P1, P2, and P3 was investigated. The zonal wind stress divergence (du/dx; Fig. 7a–c) and the meridional wind stress divergence (dv/dy; Fig. 7d–f) had similar annual variations considering their evolution: a positive signal west of 160°W starting from January to April; a negative component extending westwards throughout the year; and another positive component dominating around 160°W–100°W from June to September. The Ekman upwelling in our weighted theory was jointly caused by the zonal and meridional wind stress divergence; moreover, the meridional wind stress divergence appeared to primarily contribute in terms of its strength. Considering the difference map for P3 and P1 (Fig. 7g–h), the pattern of the meridional wind stress divergence with intermodel consistency greater than 70% positioned in the western equatorial Pacific strongly corresponded, in particular, with that of the Ekman upwelling in the west (Fig. 5o), while the zonal wind stress divergence revealed a consistent but relatively minor signal. Hence, we believe that a gradual change in the Ekman upwelling over these 90 years is chiefly attributable to the meridional surface wind stress divergence, and that both the positive signal at the beginning of the year and the prevailing negative component throughout the year weakened particularly over the central and western Pacific.

## Discussion and conclusions

The equatorial Pacific upwelling annual cycle contributes a crucial role in the tropical climatic system. Using the linear-weighted theory developed by Wang et al.^13^, based on the ZC ocean model, a series of studies were undertaken, demonstrating that equatorial upwelling annual cycles could be considered to be a combination of two dynamic processes: locally wind-driven Ekman process and remotely wind-driven wave process. Here, we analyzed the dynamics of the equatorial Pacific upwelling annual cycle based on the CMIP5 future projections and explored how the impacts of the Ekman and wave processes could vary under global warming.

The theoretical upwelling annual cycles corresponded well with the original one yielded in the CMIP5 future projections; however, the pattern correlation slightly reduced as compared to that of the present stage. The equatorial upwelling annual cycle pattern of the future warming projection appeared to remain similar in comparison to that of the present stage; however, the amplitude noticeably weakened. The linear-weighted theory further suggested that the weakening of the upwelling annual cycle was mainly attributable to the Ekman process west of 140°W and the wave process east of 140°W. Selected CMIP5 models indicates robust changes (with model agreement of 70%) in equatorial Pacific upwelling annual cycle under global warming.

To discern the changes in the dynamics of the upwelling annual cycle in the CMIP5 future projections, we divided the 90-year future projections into three 30-year horizons; thereafter, the contrast between these three time-horizons was analyzed to understand the long-term evolution of the upwelling annual cycles. The long-term variations during the future projections were similar to the one between the present stage and future projections, which further helped confirm the existence of an overall weakening tendency in the intensity considering the entire equatorial upwelling annual cycle under global warming. Due to the weakening of the meridional surface wind stress divergence, the Ekman upwelling gradually weakened. Moreover, the annual fluctuations in the equatorial thermocline depth gradually smoothed over most of the Pacific basin, which primarily contributed to the weakening of the wave upwelling. The weakening of the wave (Ekman) upwelling in the east (west) jointly contributed to the gradual weakening of the upwelling annual cycle in the equatorial Pacific. These expected changes are consistent across chosen CMIP5 models with intermodel consistency greater than 70%.

Besides the coupled models’ behaviors, the value we would like to highlight in this study is the successful capability of the linear-weighted theory on reproducing equatorial upwelling annual cycles through the combination of Ekman (*We*) and wave (*Wh*) dynamics. With these analyses, we believe that this theory could help us reproduce the equatorial upwelling annual cycle patterns that are closest to real ones, especially when we don’t have enough observed or modeled information. There are two key advantages of pairing CMIP5 simulations (original upwelling) and the linear-weighted theory result (theoretical upwelling) together: 1. Via the comparison between the original and the theoretical results, we are able to further find out which dynamics (Ekman or wave process) is more responsible for the long-term change of the equatorial upwelling annual cycle under climate variability. 2. In the case that the original w doesn’t work that well due to some reason, the theory could cover the limitation and help provide us the patterns based on the Ekman and wave dynamics.

The long-term variations in the equatorial upwelling annual cycle are related to the cold tongue evolution and variability in the marine ecosystem; moreover, they also greatly impact significant climate phenomena originating from the tropics with strong seasonal-locking features, e.g., ENSO and PNA teleconnections. The equatorial upwelling pumps cold water from the subsurface ocean layer to the surface layer and affects the background SST variations closely related to the variability of the convection, precipitation, and atmosphere–ocean interactions in the tropics; hence, the weakening of the equatorial upwelling annual cycle under global warming plays an essential role in the long-term variations in the ENSO and PNA evolutions by modifying the background strength of their mature phases. Clarifying the evolutions of the equatorial upwelling annual cycle is necessary to better understand how such significant climatic interannual phenomena could evolve in the future.

## Methods

19 CMIP5 model simulations were selected to investigate the equatorial upwelling dynamics under global warming. The selected models are the same as those in Wang et al.^14^ and Wang and Yu^15^. However, we excluded some models that do not provide data with RCP 8.5 warming scenario. The relevant model descriptions and experiment design are described in detail in Taylor et al.^22^. The Intergovernmental Panel on Climate Change (IPCC) fifth assessment report (AR5) RCP 8.5 emission scenario includes assumptions about high population and relatively slow income growth with a low rate of technological and energy intensity development; for a long time, this could lead to high energy demand and greenhouse gas emissions in the absence of climate change policies. The RCP 8.5 represents a high greenhouse gas emissions pathway as compared to the scenario literature^23,24^. In this scenario, the level of radiative forcing reaches 8.5 W/m^2^ at the end of the century characterized by an increase greenhouse gas emissions over time.

A series of historical simulations for the twentieth century (1976–2005) and RCP 8.5 emission scenario projections for the twenty-first century (2071–2100) based on the 19 CMIP5 models were analyzed here. In addition, data of the 90 years (2011–2100) under the RCP 8.5 warming scenario was utilized to examine the dynamics of variability in the upwelling annual cycle. We primarily focused on the outcome of the CMIP5 MME in order to reduce errors related intermodel variability. The performance of each model has also been discussed. The intermodel consistency calculated as the ratio of the number of models projecting the same sign as the MME to the total number of models was used to examine the robustness of the projected MME results. For the intermodel consistency reaches the 5% level of significance, more than 70% of models need to have the same change of sign, based on a binomial distribution^25,26^. Following Obilor and Amadi^27^, the Student’s t-distribution was used to assess the significance of all correlation coefficients. All model data was resampled onto a 1° latitude × 1° longitude grid. Detailed description about the selected models is summarized in Table 1. The observed vertical velocity is obtained from the European Centre for Medium-Range Weather Forecasts (ECMWF) operational ocean analysis/reanalysis system (ORA-S3). With its high spatial resolution, ORA-S3 has been used in many previous studies to examine the dynamics of equatorial upwelling annual cycle^13–15^. This research defined the annual cycles as the anomalies with respect to the climate annual mean of the entire period.

**Table 1:** List of the CMIP5 models analyzed in the study.

Model name	Modeling center
ACCESS1-0	The Centre for Australian Weather and Climate Research, Australia
ACCESS1-3	The Centre for Australian Weather and Climate Research, Australia
bcc-csm1-1	Beijing Climate Center, China Meteorological Administration, China
bcc-csm1-1-m	Beijing Climate Center, China Meteorological Administration, China
CCSM4	National Center for Atmospheric Research, US
CESM1-BGC	National Center for Atmospheric Research, US
CESM1-CAM5	National Center for Atmospheric Research, US
CMCC-CM	Centro Euro-Mediterraneo per I Cambiamenti Climatici, Italy
CMCC-CMS	Centro Euro-Mediterraneo per I Cambiamenti Climatici, Italy
FGOALS-g2	LASG, Institute of Atmospheric Physics, Chinese Academy of Sciences, China
GFDL-ESM2M	NOAA Geophysical Fluid Dynamics Laboratory, US
GISS-E2-R	NASA Goddard Institute for Space Studies, US
HadGEM2-CC	Met Office Hadley Centre, UK
HadGEM2-ES	Met Office Hadley Centre, UK
IPSL-CM5A-LR	Institut Pierre-Simon Laplace, France
IPSL-CM5A-MR	Institut Pierre-Simon Laplace, France
IPSL-CM5B-LR	Institut Pierre-Simon Laplace, France
NorESM1-M	Norwegian Climate Centre, Norway
NorESM1-ME	Norwegian Climate Centre, Norway

To discern the dynamics of the equatorial upwelling annual cycle, we applied the weighted theory derived by Wang et al.^13^ based on the ZC ocean model. The nonlocal wind-driven wave upwelling (*W*_*h*_) and local wind-driven Ekman upwelling (*W*_*e*_) and are linearly combined.

1 $${\text{w}} = w_{e} \cdot \left[ {1 - R\left( x \right)} \right] + w_{h} \cdot R\left( x \right)$$

2 $$R(x) = H_{1} /H(x) = 50/H(x) = 1\quad {\text{if}}\,\,{\text{H}}(x) < 50$$

where *W*_*e*_ was calculated from the surface wind stress, while *W*_*h*_ was calculated from the vertical displacement of the thermocline. Here, *H* and *H*_*1*_ represent the depths of the mean climatic thermocline and the constant mixed layer, respectively. The main variables generally followed the definition of the ZC ocean model, except that here, the weighting function R(x), which varied with longitude, has been considered in the weighted theory to characterize the important drivers of the equatorial upwelling annual cycle. Previous studies pointed out that the weighted theory works well in characterizing the dynamics of equatorial upwelling annual cycle over the Pacific and Atlantic Oceans in observations as well as CMIP5 simulations^13–15^. The supplemental information provides further details on the weighted theory.

## Supplementary Information

Supplementary Information.

## Data Availability

The datasets generated during the current study are available from https://esgf-node.llnl.gov/search/cmip5/. The list of the CMIP5 models analyzed in the study can be found in Table 1. The observational data ORA-S3 are available from http://apdrc.soest.hawaii.edu/data/data.php.

## Code availability

The NCL and MATLAB codes used for the calculations and creation of figures discussed in this work have been archived and are available on request from the corresponding author, L.-C. W. (lcwang@atm.ncu.edu.tw).

## References

[1] Fang CF, Wu LX (2008). The role of ocean dynamics in tropical Pacific SST response to warm climate in a fully coupled GCM. Geophys. Res. Lett..

[2] Terada M, Minobe S, Deutsch C (2020). Mechanisms of future change in equatorial upwelling: CMIP5 intermodel analysis. J. Clim..

[3] An SI, Kim JW, Im SH, Kim BM, Park JH (2012). Recent and future sea surface temperature trends in tropical Pacific warm pool and cold tongue regions. Clim. Dyn..

[4] Clement AC, Seager R, Cane M, Zebiak SE (1997). An ocean dynamical thermostat. Oceamogr. Lit. Rev..

[5] Dandonneau Y (2004). Seasonal and interannual variability of ocean color and composition of phytoplankton communities in the North Atlantic, equatorial Pacific and South Pacific. Deep Sea Res..

[6] Ding H, Keenlyside NS, Latif M (2009). Seasonal cycle in the upper equatorial Atlantic Ocean. J. Geophys. Res. Oceans.

[7] Giordani H, Caniaux G (2011). Diagnosing vertical motion in the Equatorial Atlantic. Ocean Dyn..

[8] Hagos SM, Cook KH (2009). Development of a coupled regional model and its application to the study of interactions between the West African monsoon and the eastern tropical Atlantic Ocean. J. Clim..

[9] Li T, Philander SGH (1997). On the seasonal cycle of the equatorial Atlantic Ocean. J. Clim..

[10] Richter I, Xie SP (2008). On the origin of equatorial Atlantic biases in coupled general circulation models. Clim. Dyn..

[11] Weingartner TJ, Weisberg RH (1991). On the annual cycle of equatorial upwelling in the central Atlantic Ocean. J. Phys. Oceanogr..

[12] Zebiak SE, Cane MA (1987). A model El Niño-southern oscillation. Mon. Weather Rev..

[13] Wang LC, Jin FF, Wu CR, Hsu HH (2017). Dynamics of upwelling annual cycle in the equatorial Atlantic Ocean. Geophys. Res. Lett..

[14] Wang LC, Jin FF, Wu CR (2017). Dynamics of simulated Atlantic upwelling annual cycle in CMIP5 models. J. Geophys. Res. Oceans..

[15] Wang LC, Yu JY (2019). Dynamics of upwelling annual cycle in the equatorial Pacific ocean. Sustainability.

[16] DiNezio PN (2009). Climate response of the equatorial Pacific to global warming. J. Clim..

[17] Seo H, Xie SP (2011). Response and impact of equatorial ocean dynamics and tropical instability waves in the tropical Atlantic under global warming: A regional coupled downscaling study. J. Geophys. Res. Oceans.

[18] Philander SGH (1990). El Niño, La Niña, and the Southern Oscillation.

[19] Wallace JM, Gutzler DS (1981). Teleconnections in the geopotential height field during the Northern Hemisphere winter. Mon. Weather Rev..

[20] Muñoz E, Kirtman B, Weijer W (2011). Varied representation of the Atlantic meridional overturning across multidecadal ocean reanalyses. Deep Sea Res. II.

[21] Voldoire A, Claudon M, Caniaux G, Giordani H, Roehrig R (2014). Are atmospheric biases responsible for the tropical Atlantic SST biases in the CNRM-CM5 coupled model?. Clim. Dyn..

[22] Taylor KE, Stouffer RJ, Meehl GA (2012). An overview of CMIP5 and the experiment design. Bull. Am. Meteorol. Soc..

[23] Fisher, B. S. *et al.* Issues related to mitigation in the long term context. In *Climate Change 2007: Mitigation. Contribution of Working Group III to the Fourth Assessment Report of the Inter-governmental Panel on Climate Change* (eds Metz, B., Davidson, O. R., Bosch, P. R., Dave, R. & Meyer, L. A.) (Cambridge University Press, 2007).

[24] IPCC. *Towards New Scenarios for Analysis of Emissions, Climate Change, Impacts, and Response Strategies*. IPCC Expert Meeting Report on New Scenarios, Noordwijkerhout, Intergovernmental Panel on Climate Change (2008).

[25] Power SB, Delage F, Colman R, Moise A (2012). Consensus on twenty-first-century rainfall projections in climate models more widespread than previously thought. J. Clim..

[26] Xu K, Tam CY, Zhu C, Liu B, Wang W (2017). CMIP5 projections of two types of El Niño and their related tropical precipitation in the twenty-first century. J. Clim..

[27] Obilor EI, Amadi EC (2018). Test for significance of Pearson’s correla-tion coefficient. Int. J. Innov. Math Stat. Energy Policies.

