# Supplementary material for: Continued weakening of the equatorial Pacific upwelling annual cycle in CMIP5 future projections
Source: Sci Rep. 2022 Sep 16;12:15595. doi: 10.1038/s41598-022-19874-2 (PMC9481639; doi:10.1038/s41598-022-19874-2)
Supplement: Supplementary file 1 — Supplementary Information. [file 41598_2022_19874_MOESM1_ESM.docx]

**Supplementary material**

1. **A simple theory of the equatorial upwelling**

The governing equations for the ZC ocean model consists of the reduced-gravity model for the vertical averaged ocean current (u,v) above the thermocline together with an imbedded constant depth mixed layer. The reduced-gravity model is as below:

$u_{t}-\beta_{0}yv=-g^{'}h_{x}+\tau_{x}/\rho H$ (S1)

$\beta_{0}yu=-g^{'}h_{y}+\tau_{y}/\rho H$ (S2)

$h_{t}+H\left( u_{x}+v_{y} \right)=0$ (S3)

$Hu=H_{1}u_{1}+H_{2}u_{2}$ (S4)

where the subscripts 1 and 2 refer to the current in the mixed layer and the underlying layer, respectively; ρ (rho) is the ocean density; τ (tau) is the surface wind stress; and H and H1 refer to the depth of the climate-mean thermocline and the constant mixed layer, respectively. This reduced-gravity model equation is the same as that in the ZC model, except that the weak linear friction was ignored for consistency as we used the model code of McGregor et al.^1^ for simulations of wave upwelling.

The equations governing the shear (with subscripts) between layers 1 and 2 are:

$r_{s}u_{s}-\beta_{0}yu_{s}=\tau_{x}/\rho H_{1}$ (S5)

$r_{s}v_{s}-\beta_{0}yv_{s}=\tau_{y}/\rho H_{1}$ (S6)

$\boldsymbol{u}_{\boldsymbol{s}}=\boldsymbol{u}_{\boldsymbol{1}}-\boldsymbol{u}_{\boldsymbol{2}}$ (S7)

where a strong momentum mixing between the two layers is represented by a strong frictional coefficient r_s_=(2 day)^-1^.

The upwelling velocity could then be derived as:

$W_{1}=H_{1}\left[ \left( u_{1} \right)_{x}+\left( v_{1} \right)_{y} \right]$

$=\frac{H_{1}}{H}\cdot H\left[ u_{x}+v_{y} \right]+\frac{H_{2}}{H}\cdot H_{1}[\left( u_{s} \right)_{x}+\left( v_{s} \right)_{y}]$

$=\frac{H_{1}}{H}\cdot W_{h}+(1-\frac{H_{1}}{H})\cdot W_{e}$ (S8)

Here, instead of assuming H as a constant, we took the zonal variations in the climate-mean of the thermocline depth into consideration and thus defined the vertical velocity at 50 m as:

$W =\left( 1-R\left( x \right) \right)\cdot W_{e}+R\left( x \right)\cdot W_{h}$ (S9)

where R(x) represents the weighting function that depends on the longitude (Fig. S1). The Ekman pumping upwelling was defined the same way as in the ZC model as follows:

$w_{e}=H_{1}\left[ {(u_{s})}_{x}+{{(v}_{s})}_{y} \right]$ (S10)


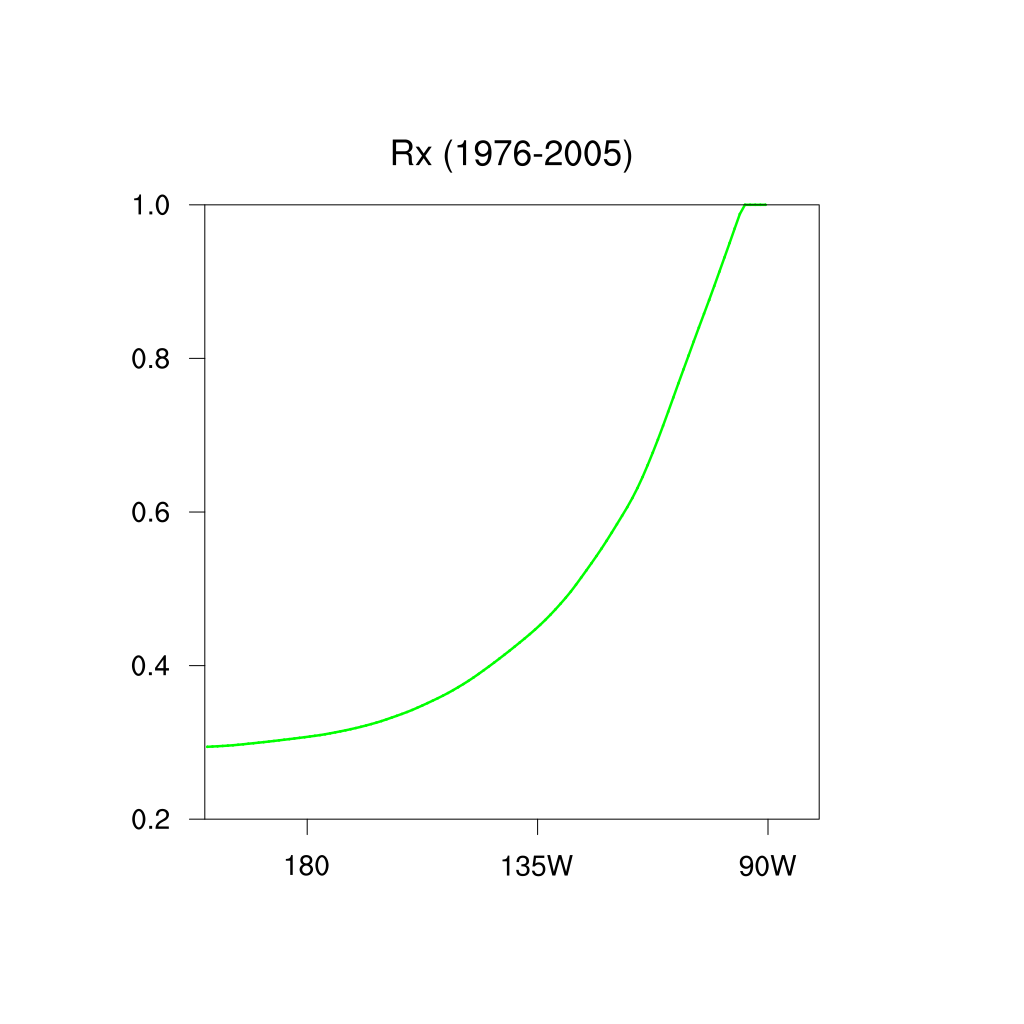


**Figure S1.** The weighting function R(x) used to estimate the equatorial Pacific upwelling annual cycle from 1976-2005. The figure was generated with The Matrix Laboratory (MATLAB) Version R2017a (<https://www.mathworks.com/> ).

1. **Validation of equatorial Pacific upwelling annual cycles in CMIP5 simulations.**

Figure S2 compares the upwelling annual cycles in the equatorial Pacific between the observations and CMIP5 simulations for the period 1976-2005. Observationally, the original equatorial upwelling annual cycle was found to be dominated by a strong component expanding all over the year in the central to eastern Pacific. The downwelling peaked around May-June, while the upwelling occurred at the beginning and the end of the year. These features were both captured by the theoretically estimated W (Fig. S2b). The pattern correlation between the original and theoretical estimated vertical velocity reached 0.8. The observed upwelling annual cycle is dominated by the Ekman upwelling (Fig. S2c), while the wave upwelling (Fig. S2d) is confined near the eastern boundary with small contribution. The pattern correlation between the original and the Ekman/wave upwelling is 0.69/0.26, respectively. Despite the weaker amplitude, the simulated original upwelling still reproduces key features of the observed upwelling. Although the contribution of Ekman and wave upwelling to theoretical upwelling is slightly different compared to the observations, the pattern correlation between the original and theoretical-reconstructed upwelling from 19 CMIP5 multi-model ensemble still reaches 0.72. This indicates that the weighted theory also works well in characterizing the equatorial Pacific upwelling annual cycle in model simulation.


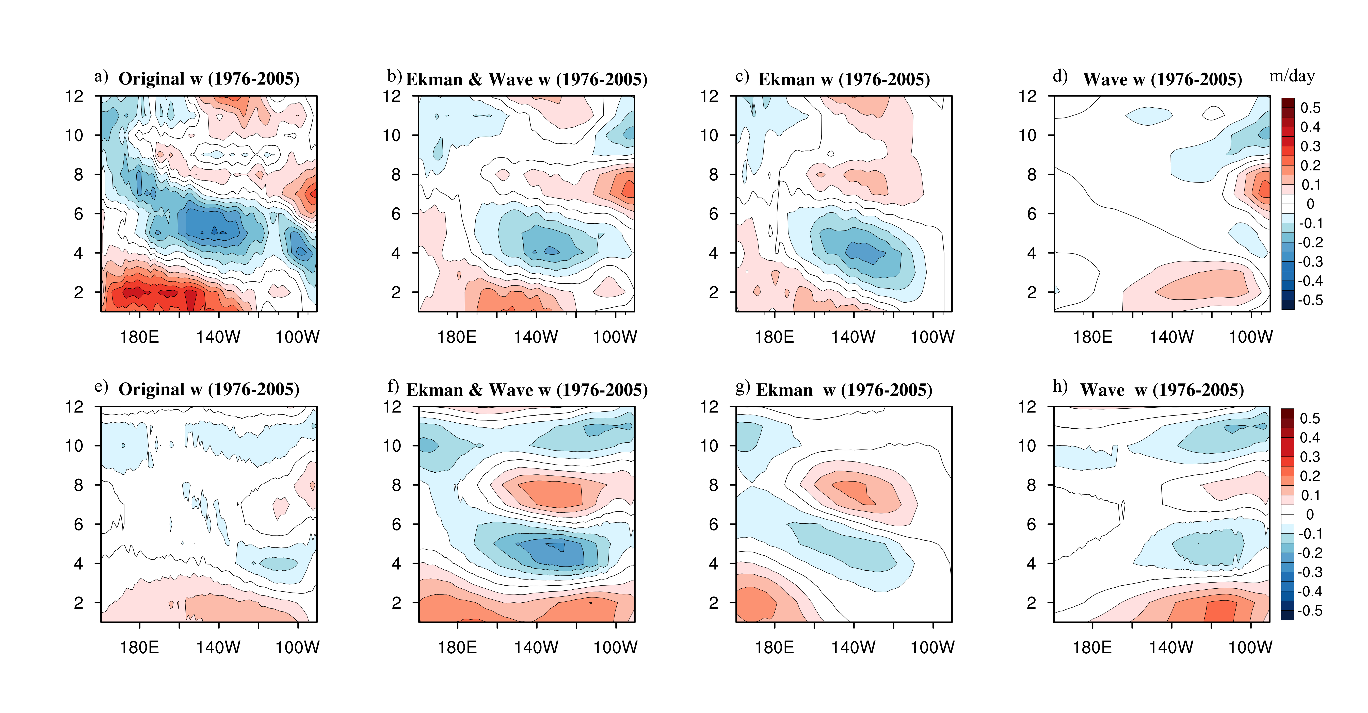


**Figure S2**: Time-longitude diagrams of the annual cycles of the (a,e) original, (b,f) theoretical, (c,g) Ekman and (d,h) wave upwellings in the equatorial Pacific averaged over 3°N-3°S and derived via observation (first row) and the CMIP5 multi-model ensemble (second row), respectively (unit: m/d). The maps were generated with NCAR Command Language (NCL) Version 6.6.2 (<http://www.ncl.ucar.edu/> ).

1. **Simulated upwelling annual cycles.**

Figure S3 shows the difference of the original upwelling annual cycles in the equatorial Pacific Ocean between the present stage and future projection. Some models projected a significant weakening of original upwelling (e.g. (a) ACCESS1-0, (b) ACCESS1-3, (j) FGOALS-G2, (m) GISS-E2-R, (r) NorESM1-M and (s) NorESM1-ME), while some models only demonstrate a small change in amplitude (e.g. (c) BCC-CSM1-1, (d) BCC_CSM1-1-m, (e) CCSM4, (h) CMCC_CM). Despite the projected amplitude difference, majority of CMIP5 models projected a weakening tendency in the equatorial Pacific upwelling in the future. Both original basin-wide enhancing pattern at the beginning of the year and the weakening pattern west of 140°W starting from April till the end of the year are projected to weaken. The semi-annual harmonic signal at the eastern Pacific is also expected to decrease in most of CMIP5 model projections. The results are consistent with CMIP5 multi-model ensemble mean results presented in the main text, further confirming a robust weakening of equatorial Pacific annual cycles in the future.


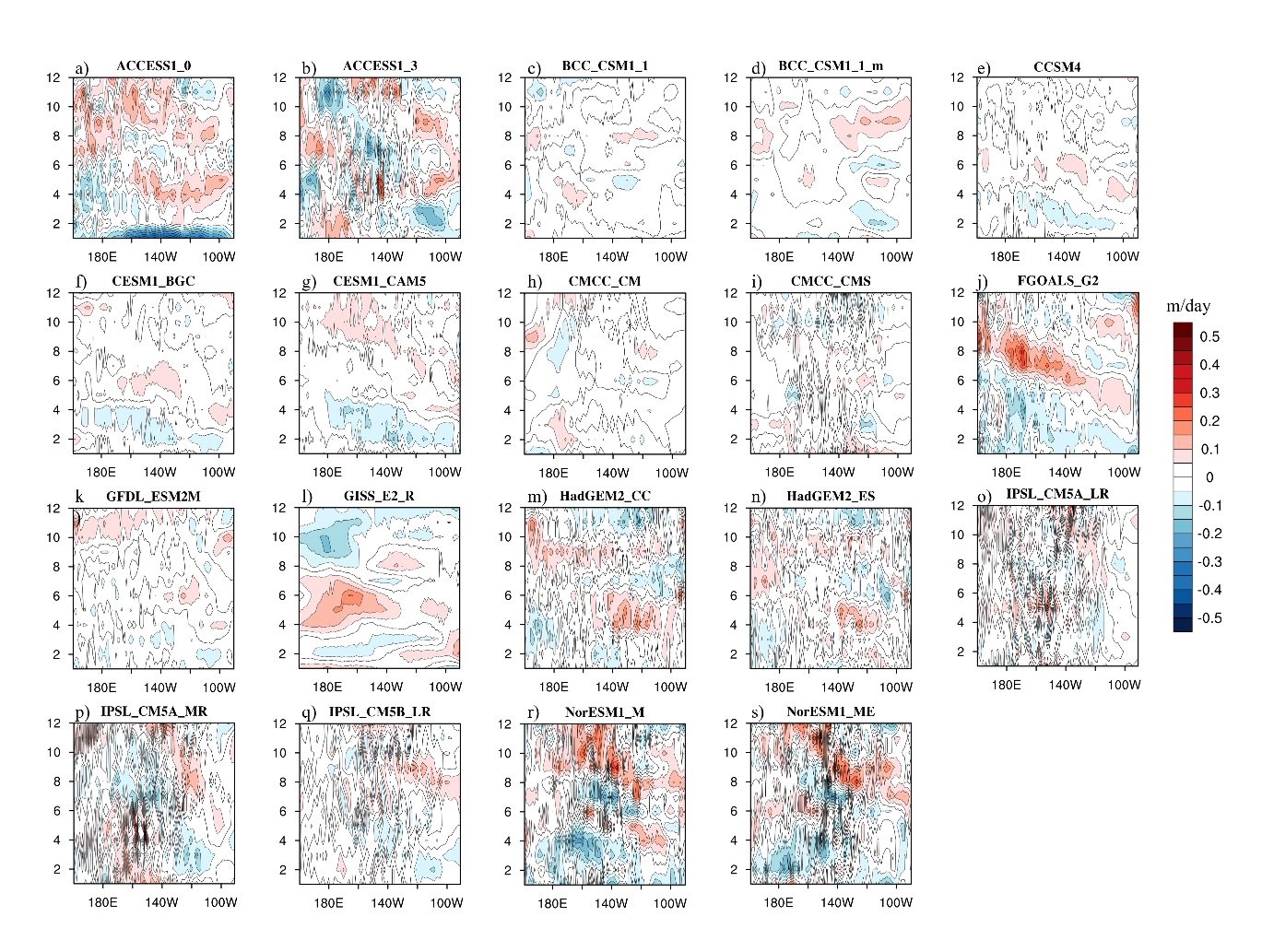


**Figure S3**: The difference of the original upwelling in the equatorial Pacific between the present stage and the future projection simulated by 19 CMIP5 models (averaged over 3°N-3°S; units: m/day). The maps were generated with NCAR Command Language (NCL) Version 6.6.2 (<http://www.ncl.ucar.edu/>).

**References**

1. McGregor, S., Holbrook, N. J. & Power, S. B. Interdecadal sea surface temperature variability in the equatorial Pacific Ocean, Part I: The role of off-equatorial wind stresses and oceanic Rossby waves, *J. Climate,* **20(11)**, 2643–2658, DOI: <https://doi.org/10.1175/JCLI4145.1> (2007).
